# Identification of key biomarkers and related immune cell infiltration in cervical cancer tissue based on bioinformatics analysis

**DOI:** 10.1038/s41598-023-37346-z

**Published:** 2023-06-21

**Authors:** Guang Zhu, Zhihui Xiong, Wenzeng Chen, Zhen Zhu, Wei Wang

**Affiliations:** Department of Obstetrics, Tongde Hospital of Zhejiang Province, No. 234, Gucui Road, Xihu District, Hangzhou, 310012 China

**Keywords:** Biotechnology, Cancer

## Abstract

Cervical cancer (CC) is the most common gynecological malignant tumor. Immunotherapy has become a new model for the treatment of CC, especially advanced and recurrent cancer. At present, many studies are exploring the safety and efficacy of immunotherapy for advanced or recurrent CC. In this study, CIBERSORT was used to analyze the immune cell infiltration in CC patients, to evaluate the proportion of immune cell types in CC samples, to quantify the cell composition of the immune response, and to analyze its prognostic value. The expression profile datasets of CC were downloaded from the GEO. The differentially expressed genes (DEGs) between CC and normal cervical tissues were identified via R software (version 4.1.1), and their functions and pathways were enriched and analyzed. A protein–protein interaction network was constructed to screen the hub gene. Immune cell infiltration in CC was analyzed via scientific reverse convolution algorithm (CIBERSORT), and the hub gene was analyzed via survival analysis to screen the diagnostic biomarkers of CC. A total of 144 DEGs and 12 hub genes were identified. DEGs are mainly involved in molecular functions such as serine-peptidase activity, serine-hydrolase activity, and chemokine activity. The enrichment pathway is closely related to the interaction between viral proteins and cytokines and cytokine receptors, the interleukin 17 signaling pathway, and chemokine signaling pathway. The immune cell infiltration analysis showed that T cells were the main infiltrating immune cells in CC, especially T cells CD8+ and CD4+ . The survival analysis of the hub gene showed that CEP55, MCM2, RFC4, and RRM2 had high diagnostic value. CEP55, MCM2, RFC4, and RRM2 can be used as diagnostic markers for CC. CD8+ and CD4+ T cells are closely related to the occurrence and development of CC.

## Introduction

Cervical cancer (CC) is one of the most common gynecologic malignancies in the clinical setting, and its incidence rate ranks fourth in female malignant tumors^1^. According to statistics by the World Health Organization, the incidence and death rates of CC are > 500 000/year and > 10 000/year, respectively^2^. The local lesions of early CC can be cured through surgery, radiotherapy, or chemotherapy. For locally advanced and metastatic cases, the effect of the above treatment methods is not satisfactory, and the 5-year survival rate is only 57.1% and 17.3%, respectively^3^. The survival rate in advanced and recurrent cases is even lower. Therefore, the study of new treatment models is important for improving the prognosis of patients with advanced and recurrent CC.

At present, with the rapid development of molecular cell biology and immunology, immunotherapy has gradually become a new treatment modality for CC^4^. More evidence shows that tumor immune cell infiltration is closely related to the occurrence and development of cancers^5^. The type and proportion of immune cell infiltration in tumors are closely related to clinical results. They not only predict the survival of patients but also affect tumor treatment. Therefore, it is expected to become a drug target and clinical biomarker^6^. The 2019 clinical practice guidelines for CC (version 1), published by the national comprehensive cancer network (NCCN), recommend that pembrolizumab be used for the second-line treatment of recurrent or metastatic CC with programmed death protein 1 ligand (PD-L1) positive or deficient mismatch repair/microsatellite instability-high (2A)^7^. On June 12, 2018, the US Food and Drug Administration approved the use of programmed cell death1 (PD-1) inhibitor, pembrolizumab (trade name: Keytruda), for the treatment of advanced and recurrent CC^8^.

In this study, the expression profile datasets of CC were downloaded from the GEO and TCGA databases, the differentially expressed genes (DEGs) were identified by R software, and the function and pathway enrichment of DEGs were analyzed. By constructing a protein–protein interaction (PPI) network, selecting key (hub) genes, drawing an SURVIVE curve, and analyzing the diagnostic value of the hub gene, we screened the diagnostic biomarkers of CC. CIBERSORT is a tool used to deconvolute the expression matrix of immune cell subtypes based on the principle of linear support vector regression. It can use the data from RNA SEQ to predict the infiltration of immune cells^9^. In this study, CIBERSORT was used to analyze the immune cell infiltration in CC patients, to evaluate the proportion of immune cell types in CC samples, to quantify the cell composition of the immune response, and to analyze its prognostic value.

## Methods

### Materials

The expression profile datasets of five CC were downloaded from the GEO database: GSE 7410, GSE 9750, GSE 14,404, GSE 63,514, and GSE 63,678, respectively. The selection criteria were (1) normal cervical tissue and CC tissue samples, (2) the test type is expression profiling by array, and (3) species selection “Homo sapiens.” The chip platform note files were GPL 1708, GPL 96, GPL 6699, GPL 570, and GPL 571 (Table S1). The name of the microarray probe was converted into the gene symbol via Perl (version 5.30), whereas the DEGs were screened by the R language limma software package between normal cervical samples and CC samples in each expression dataset. The screening standard was | log2 fold change (FC) |> 1, whereas the corrected *p* value was < 0.05. Then the common targets of these five datasets were compared and analyzed with Venn R, and the intersection targets of five datasets were displayed through a Venn diagram.

### Robust rank aggregation (RRA) analysis

The RRA method involves integrating ranking lists using a probability model^10^. Some studies have used it to integrate gene lists of multiple sets of chip data and achieved positive results^11^. To integrate five microarray datasets, this study used the RRA method to determine robust DEGs. First, the up-regulated and down-regulated genes of each dataset were obtained according to the FC ranking; then, through the RRA R software package, robust DEGs were obtained according to the sequencing genes in five datasets, and they were statistically significant, with | log2 FC |> 1 and *p-*value < 0.05 as the standard.

### Gene ontology (GO) and Kyoto encyclopedia of genes and genomes (KEGG) pathway enrichment analysis

To determine the functional role of the above core DEGs, the GO enrichment results of biological process (BPs), cellular component (CCs), and molecular function (MFs) were obtained through the R software package “clusterprofiler”^12,13^. The KEGG pathway of DEGs was analyzed via R software package, and the difference was statistically significant (*p* < 0.05)^14,15^.

### Protein–protein interaction network (PPI network) construction and module analysis

The above robust DEGs were uploaded to the online database of STRING to construct and clarify the PPI network^16^. The species was selected as “Homo sapiens,” the threshold was selected as “highest confidence,” and the unconnected nodes were hidden in the network. The target PPI relationship data column was obtained and saved as a TSV format file. The protein interaction network graphic visualization PPI network was obtained and executed via Cytoscape (version 3.8.2)^17^.

The above TSV file data were imported into Cytoscape software, and the MCODE cluster plug-in^18^ was used to build key network modules. Each network module used annotation, visualization, and R language to conduct the GO biological process and signal pathway enrichment analysis of KEGG for the target genes contained in the key network modules. The difference was statistically significant, with *p* < 0.05.

### Hub gene screening

CytoHubba is a plug-in of Cytoscape and provides several topological analysis algorithms^19^, including Degree, Edge Percolated Component (EPC), Maximum Neighborhood Component (MNC), Density of Maximum Neighborhood Component (DMNC), Maximal Clique Centrality (MCC), and six centralities, including BottleNeck, EcCentricity, Closeness, Radiality, Betweenness, and Stress. These algorithms can be used to screen for hub genes.

### CIBERSORT immune infiltration analysis

The CIBERSORT algorithm was used to analyze the previously obtained standardized gene expression data, and the proportion of 22 immune cells was obtained^9^. The “vioplot” of R software^20^ was used to compare the infiltration level of each immune cell between the two groups to calculate the percentage of each immune cell in the sample and to conduct a principal component analysis (PCA).

### Survival analysis

RNA-seqFPKM data and prognostic information of cervical cancer patients were downloaded from the TCGA database. The survival analysis of the hub gene was analyzed via the R software package survminer^21^, The high and low levels of hub gene expression are determined by the point with the most significant split, and the difference was statistically significant, with *p* < 0.05.

## Results and discussion

### Data preprocessing and DEGs identification

**Figure 1:**
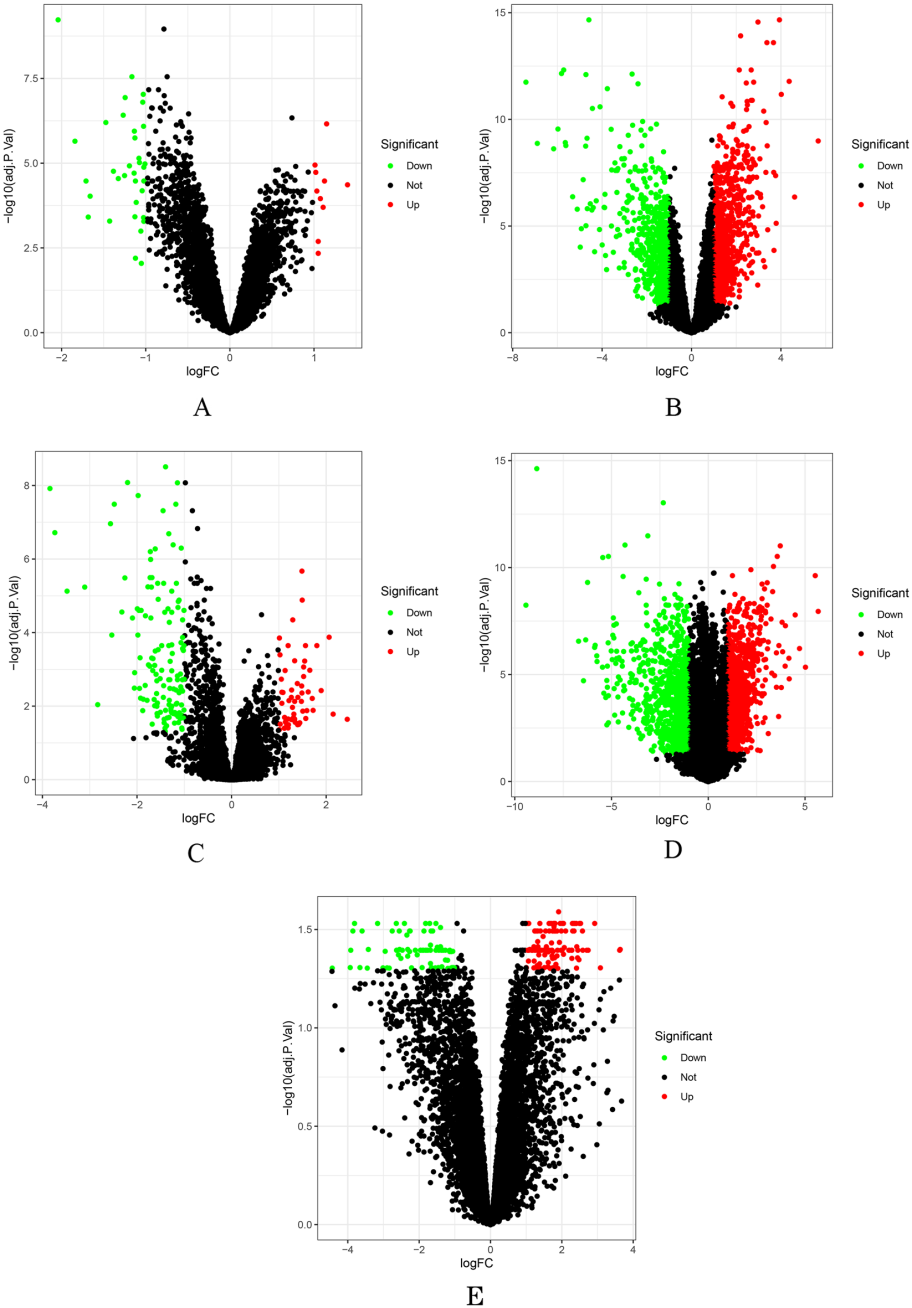
(**A–E**) Identification of DEGs and robust DEGs. Volcano plots of the distribution of DEGs in GSE 7410, (**A**), GSE 9750 (**B**), GSE 14,404 (**C**), GSE 63,514 (**D**), and GSE 63,678 (**E**). The red and green dots represent the upregulated and downregulated genes, respectively, whereas the black dots represent genes that cause no difference.

GSE 7410 studied early-stage cervical tumor (including 19 lymph node metastasis and 21 without lymph node metastasis) versus healthy cervical tissue. GSE 9750, GSE 14,404, GSE 63,514, and GSE 63,678 are cervical cancer versus normal cervical tissue. |Log2 FC |> 1 and corrected *p* value < 0.05 were used as screening criteria, and 44, 1513, 192, 1928, and 194 differential genes were obtained from GSE 7410, GSE 9750, GSE 14,404, GSE 63,514, and GSE 63,678, respectively. The up-regulated genes were 10, 823, 57, 952, and 117, whereas the down-regulated genes were 34, 690, 135, 976, and 77, respectively. The volcano map of differential genes is shown in Fig. 1A–E, in which the red and green dots represent up-regulated and down-regulated genes, respectively. Whereas the black dots represent genes that cause no difference. A Venn diagram show common genes between fives datasets (Fig. S1).

### RRA analysis

**Figure 2:**
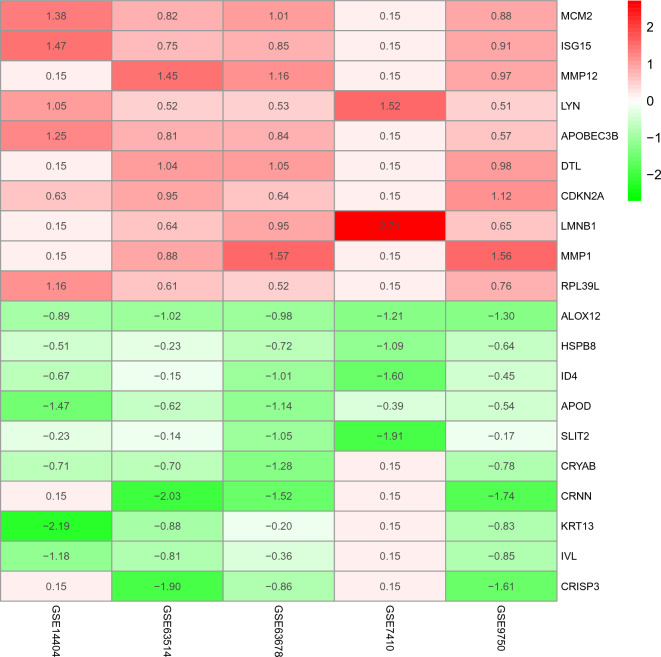
The heatmap of top 10 upregulated and downregulated robust DEGs identified by the RRA method. Red represents high expression robust DEGs, while green represents low expression robust DEGs. DEG, differentially expressed gene; RRA, robust rank aggregation.

The DEGs in the five datasets were screened via the RRA method, and a total of 144 DEGs (CC/normal cervical tissue) were obtained, including 61 up-regulated and 83 down-regulated genes. The top 10 up-regulated and down-regulated differential genes were used to make the DEGs heat map (Fig. 2).

### GO and KEGG pathway enrichment analysis

**Figure 3:**
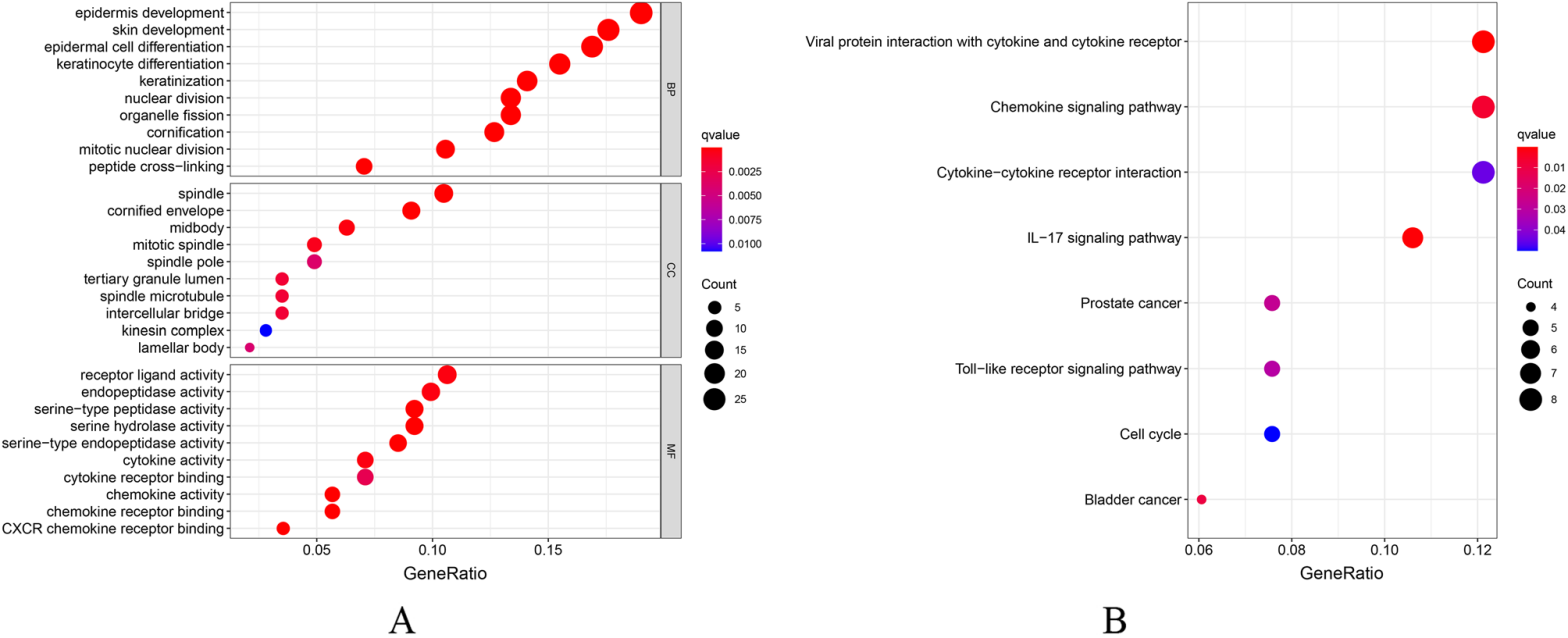
(**A**) GO analysis results of DEGs; (**B**) Results of the DEGs pathway analysis.

The results of the GO analysis showed that the biological process changes of DEGs were significantly enriched in keratinization, epidermal development, and epidermal cell differentiation, and that the changes in the cell components were mainly in the keratinized envelope, spindle, and mesosome, whereas those in the molecular function were mainly enriched in the serine-peptidase activity, serine-hydrolase activity, and chemokine activity (Fig. 3A)^13^. The results of the KEGG pathway enrichment analysis showed that the enriched pathways were mainly closely related to the interaction between viral proteins and cytokines and cytokine receptors, the interleukin-17 signaling pathway, and the chemokine signaling pathway (Fig. 3B)^14^.

### PPI network construction and module analysis

**Figure 4:**
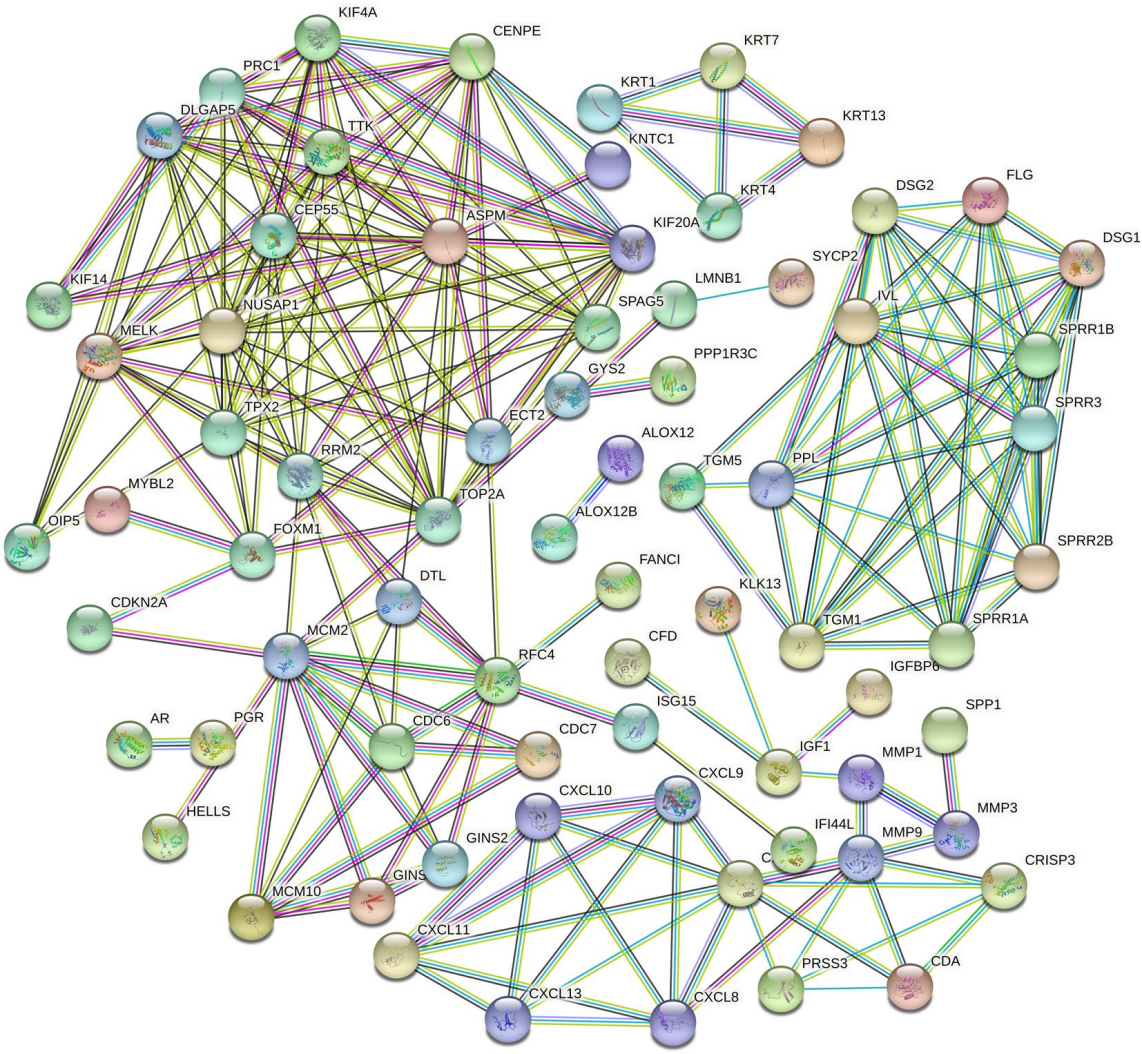
PPI network diagram. PPI network analysis graph, nodes represents proteins, thicker connection represents higher score, thicker line represents more interaction between two protein.

To further study the interaction between robust DEGs, the PPI network was constructed using STRING online database (Fig. 4)^16^. The above TSV list was imported into Cytoscape software, Cytoscape ClusterViz was used for module analysis, the MCODE algorithm was selected for the cluster analysis of robust DEGs^18^, and the default parameters, node score threshold = 0.2 and k-core threshold = 4, were set. K-core is a parameter that determines the size of the identification module—that is, the edge corresponding to the obtained module should be greater than 4. Finally, three key network modules and 34 targets were obtained, of which the largest network module was composed of 14 action node targets (Table S2). The node targets contained in these modules may be crucial nodes of CC. The targets contained in each of the above network modules were enriched and analyzed in R language, and the *p* value < 0.05 was statistically significant (Table S3).

### Hub gene selection

**Figure 5:**
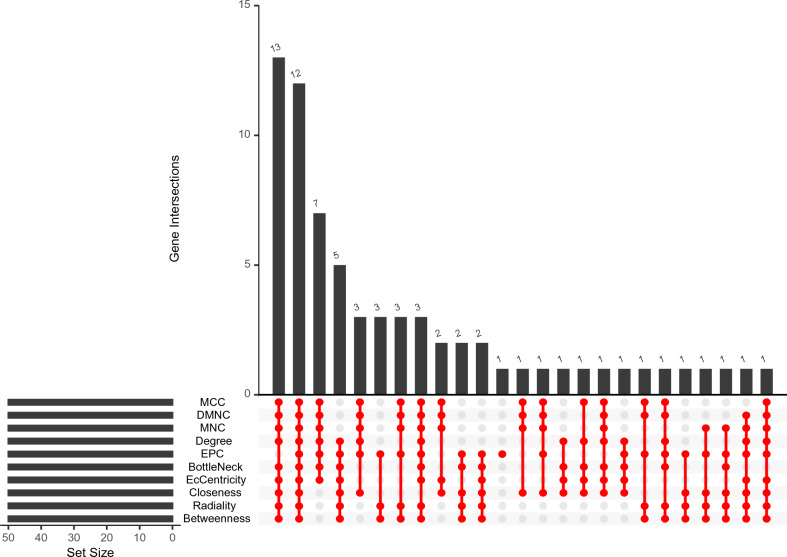
Identification of Hub genes. Hub genes were identified by intersection of 50 genes from 10 algorithms including MCC, DMNC, MNC, Degree, EPC, BottleNeck, EcCentricity, Closeness, Radiality, and Betweenness.

The Cytoscape plug-in CytoHubba contains a variety of topological analysis algorithms (MCC, DMNC, MNC, Degree, EPC, BottleNeck, EcCentricity, Closeness, Radiality, Betweenness, and Stress), which can be used to predict and explore important nodes in PPI networks. The score of the topology algorithm is assigned to each node in the PPI network. According to the gene score, the top genes are regarded as hub genes. In this study, the top 50 genes in the network were ranked by 10 topological analysis algorithms (MCC, DMNC, MNC, Degree, EPC, BottleNeck, EcCentricity, Closeness, Radiality, Betweenness) and 12 hub genes were obtained: KIF20A, TPX2, CENPE, CEP55, TOP2A, FOXM1, OIP5, RRM2, RFC4, GINS1, MMP9, and MCM2 (Fig. 5). At the same time, the descriptions of the 12 hub genes were displayed, including the full names, aliases, and main functions (Table S4).

### Immune cell infiltration analysis

**Figure 6:**
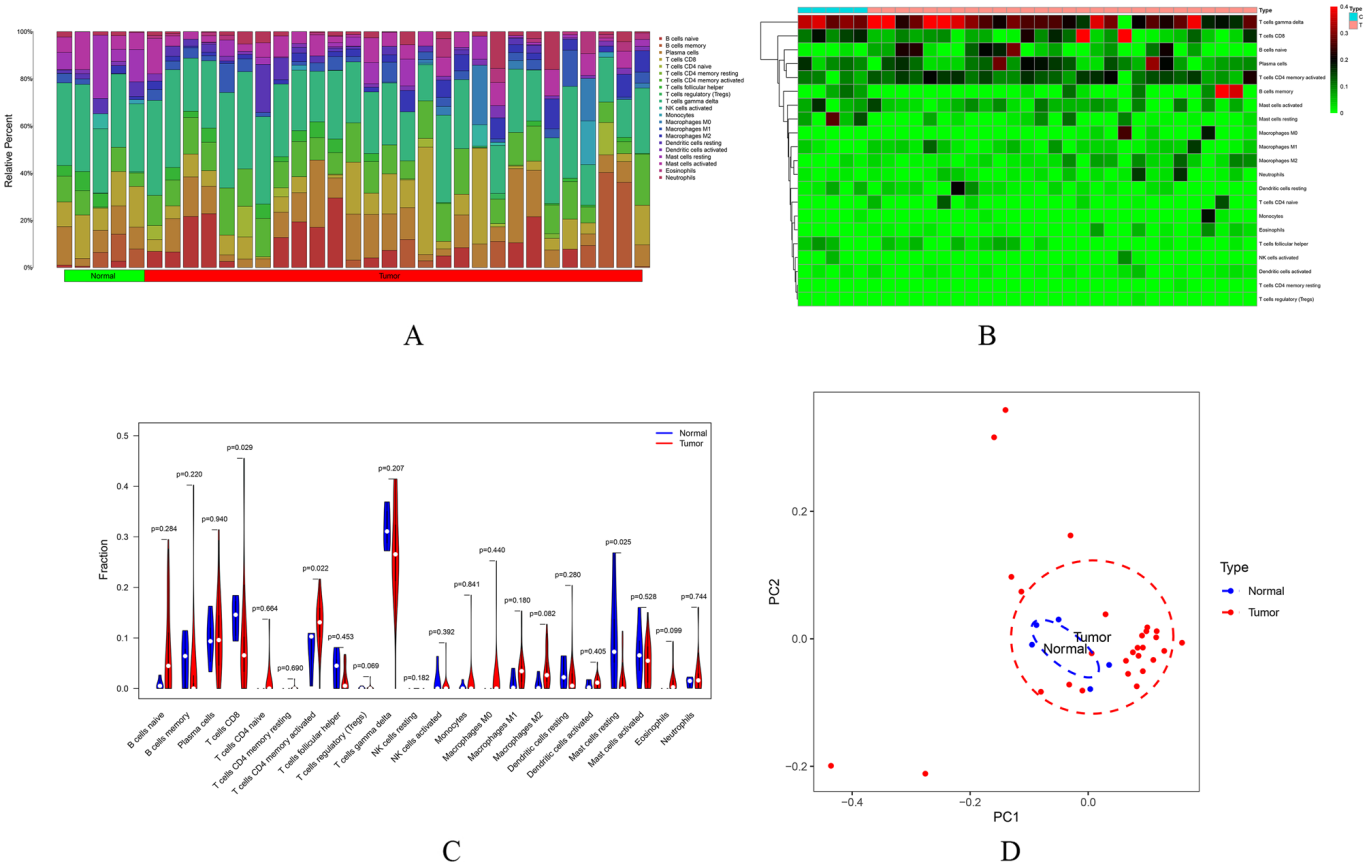
Immune cells infiltration analysis. (**A**) The distribution of 22 types of immune cells between cervical cancer and normal cervical tissues; (**B**) The difference of immune cells infiltration between cervical cancer and normal cervical tissue visualized by heatmap (*P* < 0.05); (**C**) Violin plot visualizing the differentially infiltrated immune cells (*P* < 0.05); (**D**) PCA performed on all cervical tissues. The two principal components showed nothing significant variation. PCA, principal component analysis.

Using the CIBERSORT algorithm, the infiltration of 21 immune cells in 33 CC samples is shown in Fig. 6A. There is no significant difference in immune cell infiltration between normal cervical tissue and cervical cancer tissue. However, compared with other immune cells, T cell infiltration was dominant in both normal control samples and CC samples (Fig. 6B). These results suggest T cells may play an important role in the occurrence and development of CC. A visual violin diagram was also constructed to demonstrate the above findings (Fig. 6C). The PCA in Fig. 6D shows no differences between normal controls and CC tissue samples.

### Survival analysis

**Figure 7:**
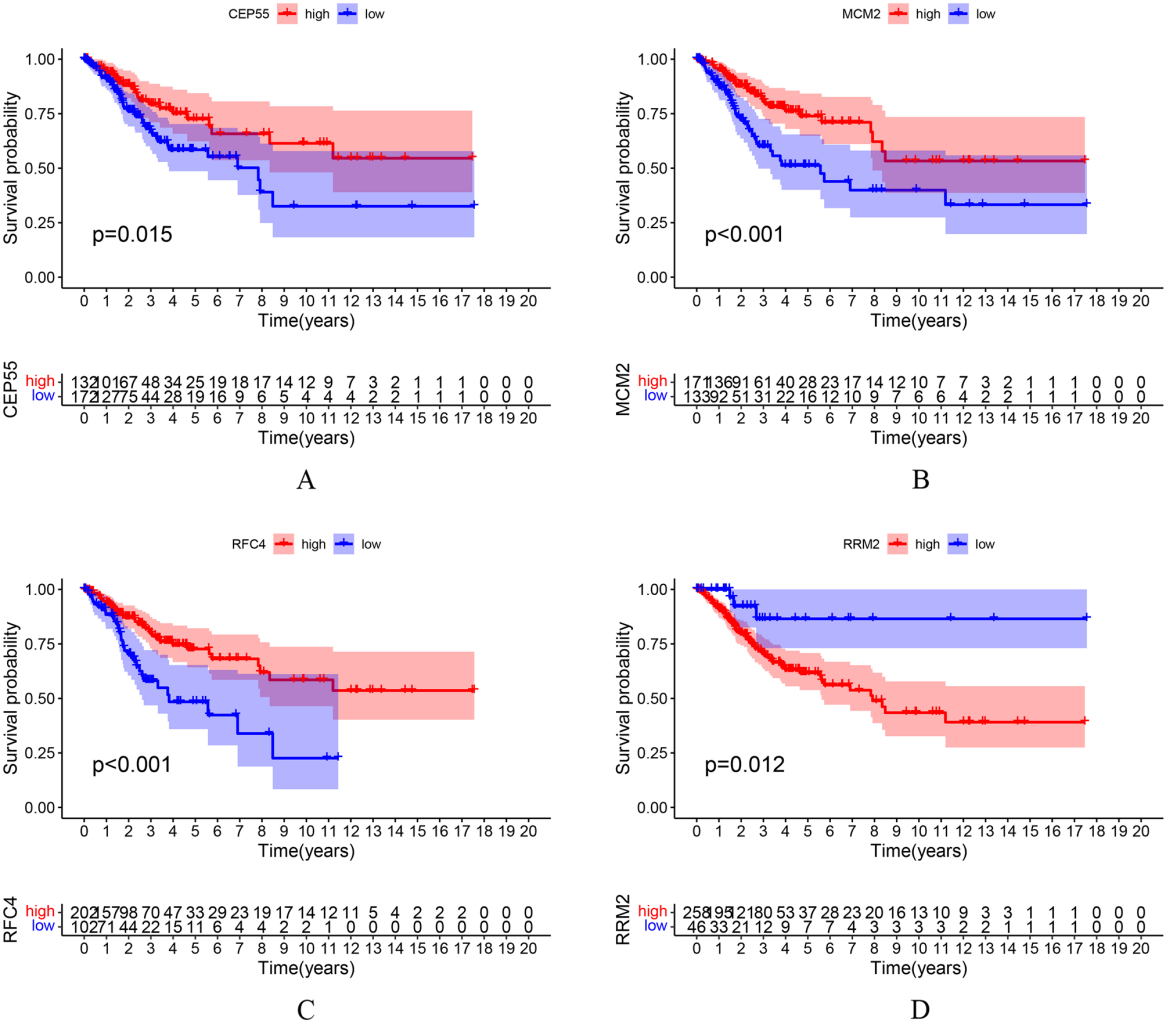
Survival analysis. Gene changes of CEP55 (**A**), MCM2 (**B**), RFC4 (**C**), and RRM2 (**D**) were significantly correlated with the overall survival of cervical cancer patients (*P* < 0.05).

The R software package was used to analyze the association between the four hub genes and the overall survival rate of CC patients. There is no statistically significant difference in age distribution between the high and low expression groups for these genes (Fig. S2). According to the best-separation cut-off value for each hub gene, the samples of CC patients were divided into two groups to obtain the Kaplan-Meier (K-M) survival curve in the target cervical cancer datasets. The results showed that the gene changes of CEP55 (*p* = 0.015), MCM2 (*P* < 0.01), RFC4 (*P* < 0.01), and RRM2 (*P* = 0.0012) were significantly correlated with the overall survival rate of patients with CC (Fig. 7).

### Discussion

Cervical cancer (CC) is one of the important cancers perplexing women all over the world. The high mortality of CC is largely due to the untimely diagnosis, so it is important to find specific biomarkers for early diagnosis to improve the prognosis of CC patients. Several studies have shown that the genes with partial disorder in CC patients can be used as biomarkers for their diagnosis^22,23^. Immunotherapy is an anti-cancer therapy that has attracted much recent attention. In the past few years, immunotherapy, such as antibody PD-1/PD-L1 signal pathway inhibitors, has made a breakthrough in the field of oncology^24^; however, there are still some problems to be solved. For example, biomarkers need to be screened before medication, and patients who meet the standards can be injected. Therefore, looking for specific diagnostic and therapeutic biomarkers is important for improving the prognosis of CC patients. In the past, immunohistochemistry or flow cytometry were mostly used to analyze the infiltration pattern of immune cells in tumor tissue. The procedure was cumbersome and the feedback efficiency was low. The CIBERSORT software used in this study was invented by Newman et al. a researcher at Stanford University School of Medicine, in 2015. The software adopted deconvolution algorithm, which can accurately and rapidly analyze the gene expression profile data of complex tissues, with obvious advantages.

In this study, we characterized genetic alteration of 133 cervical cancer cases in Caucasian cervical cancer patients in the GEO, patients’ clinical data were carefully reviewed, and 144 DEGs were identified using the bioinformatics method. GO function enrichment analysis showed that DEGs were mainly involved in biological processes such as epidermal development and skin development, mediated molecular functions such as receptor ligand activity and endopeptidase activity, and DEGs gene products were mainly enriched in the spindle, cutin envelope, and mesosome. The enriched pathway is mainly related to the interaction between viral protein and cytokines and their receptors, the chemokine signaling pathway, cytokine–cytokine receptor interaction, and the IL-17 signaling pathway. Therefore, we speculate that the abnormal activation of multiple inflammatory pathways may be related to cervical injury after human papilloma virus (HPV) infection. Chemokines widely exist in inflammatory response tissues, and they induce inflammatory cells to participate in an immune response. A large number of experimental studies show that chemokines play a critical role in the pathogenesis of CC^25^. Bai et al.^26^ found that overexpression of CXCL5 was involved in the tumor development of CC. Zhang et al.^25^ found that overexpression of akip1 in CC cells increased the levels of CXCL1, CXCL2, and CXCL8. Paradkar et al.^27^ showed that the combined diagnosis of cytokines and their receptors can more objectively reflect the condition and development of CC patients. Lv et al.^28^ found that interleukin-17A (IL-17A) is a pro-inflammatory cytokine derived from CD4 T cells, which is involved in the occurrence of human cervical tumors. The above results are consistent with the results of this study, suggesting that the accuracy of this study is high.

In addition, based on the module analysis, we found that three key modules are closely related to the pyrimidine metabolism pathway and chemokine signaling pathway. RRM2 and TOP2A are important participants in the pyrimidine metabolic pathway. Chemokines play an important role in chemokine signal transduction. RRM2 is the regulatory subunit M2 of ribonucleotide reductase, which participates in pyrimidine metabolism and other processes. This gene encodes a subunit of ribonucleic acid reductase, which catalyzes ribonucleic acid to produce deoxyribonucleic acid and affects the cell cycle^29^. Wang et al.^30^ found that RRM2 is related to apoptosis and the proliferation of CC cells, and inhibiting RRM2 expression can be used as a potential target for human CC treatment. TOP2A is topoisomerase IIA, which is considered to be closely related to the occurrence and development of CC. Wang et al.^31^ found that TOP2A is abnormally highly expressed in CC tissues through experiments, and that TOP2A leads to cell migration, invasion, and epithelial mesenchymal transformation by activating the PI3K/Akt signaling pathway. Li et al.^32^ found that HPV can activate the toll-like receptor (TLR)/nitric oxide (no) signaling pathway, which may be involved in the pathogenesis of CC caused by HR-HPV. Chemokine is one of the many factors involved in the progression from cervical intraepithelial neoplasia to CC. Whether chemokine receptor depends on the existence of immune signaling pathway to regulate the production of chemokine in CC tissue still needs to be further verified by experiments.

This study constructed the PPI network of DEGs and identified KIF20A, TPX2, CENPE, CEP55, TOP2A, FOXM1, OIP5, RRM2, RFC4, GINS1, MMP9, and MCM2 as hub genes. TPX2, CEP55, TOP2A, FoxM1, RRM2, RFC4, MMP9, and MCM2 have proven to be closely related to the grading and histological type of CC, and TPX2 is closely related to the proliferation, migration, and invasion of CC cells and the cell cycle^33^. The high expression of CEP55 in CC tissue is significantly correlated with lymph node metastasis and advanced tumor stage, which is an independent predictor of poor prognosis in CC patients^34^. The highly expressed TOP2A may participate in the occurrence, development, invasion, and metastasis of CC by regulating the expression of VEGF^35^. FoxM1 plays a key role in the occurrence, maintenance, tumor growth, invasion, angiogenesis, and metastasis of CC^36^. Su et al.^37^ RRM2 is involved in cervical carcinogenesis and predicts poor survival, and may be a potential therapeutic target including in cisplatin treatment. The expression of RRM2 in CC is related to the degree of differentiation of the disease, and it has nothing to do with the pathological type, clinical stage, or lymph node metastasis^38^. Bachtiary et al.^39^ found the expression of RFC4 in grade III CC was higher than that in grade II CC. Niu et al.^40^ found the expression of RFC4 in cervical squamous cell carcinoma was significantly higher than that in high-grade squamous intraepithelial lesions, and it was related to the progression and prognosis of CC. The expression of MMP9 is abnormally increased in CC tissues, which is closely related to cell proliferation and plays an important role in the development of the disease^41^. MCM2 may be involved in the occurrence, development, invasion, and metastasis of CC. Detecting the expression of MCM2 can be used as a basis for judging the invasion and prognosis of CC^42^. KIF20A, CENPE, OIP5, and GINS1 are closely related to the occurrence and progression of a variety of tumors; however, their relationship with CC is not clear, which is worthy of further study.

This study evaluated the diagnostic value of hub genes and found that four genes (CEP55, RRM2, MCM2, and RFC4) have high diagnostic value. CEP55, RFC4, RRM2 genes were part of 144 DEGs obtained via the RRA method, but the Fig. 2 only indicate the top 10 up-regulated and down-regulated differential genes. They have proven to be closely related to CC and may become diagnostic biomarkers of the disease.

Through the analysis of immune cell infiltration, it was found that the increased infiltration of CD8+ T lymphocytes, CD4+ memory T lymphocytes, and the decrease of resting mast cells were related to the occurrence and development of CC. CD8+ T lymphocytes are a key functional T cell subset involved in adaptive immune response. Studies have shown that the main effector of CD8+ T cells is CXCR5+CD8+ T lymphocytes^43^, which has a potential cytotoxic effect in the microenvironment formed by chronic virus infection and malignant tumors. Enhancing CD8+ T cell activation is considered to be a key point of most immunotherapy. Other research results show that CD8+ T lymphocytes are expected to play a role in the clinical treatment of CC^44^. Under normal physiological conditions, the proportion of CD4+ and CD8+ subsets of T lymphocyte subsets is maintained within a certain range, playing a role in maintaining immune system balance^45^. Cervical lesions will cause abnormal changes in CD4+ and CD8+ subsets. With the aggravation of cervical lesions, the above change trend will continue and further promote cellular immune system imbalances, resulting in the loss of immune function^46^. This study found that mast cells are multifunctional cells^47^, which can secrete a variety of bioactive substances after activation to participate in immune and inflammatory reactions. The type and number of mast cells infiltrated in malignant tumor tissues are closely related to malignant tumor differentiation, metastasis, and prognosis.

## What is new and conclusion

In conclusion, through a bioinformatics analysis of CC expression profile data, 144 DEGs and 12 hub genes were obtained. Four diagnostic biomarkers (CEP55, RRM2, MCM2, and RFC4) were obtained by survival analysis of the hub gene. In addition, immune cell infiltration analysis found that CD4+ memory T cells and CD8+ T cells may play a role in the occurrence and development of CC. Altogether, these results may promote the new understanding of molecular mechanisms and clinically related molecular targets for prognosis in cervical cancer and provide new insight into the occurrence and development of cervical cancer. As a future research direction, the research group will verify the accuracy of the results of this study at the molecular, cellular, and tissue levels and further explore the regulatory relationship between the four diagnostic markers and CD4+ memory T cells and CD8+ T cells.

## Supplementary Information

Supplementary Legends.

Supplementary Figure S1.

Supplementary Figure S2.

Supplementary Table S1.

Supplementary Table S2.

Supplementary Table S3.

Supplementary Table S4.

## Data Availability

All data generated or analysed during this study are included in this published article.
