# Supplementary material for: Identification of key biomarkers and related immune cell infiltration in cervical cancer tissue based on bioinformatics analysis
Source: Sci Rep. 2023 Jun 21;13:10121. doi: 10.1038/s41598-023-37346-z (PMC10284792; doi:10.1038/s41598-023-37346-z)
Supplement: Supplementary file 1 — Supplementary Legends. [file 41598_2023_37346_MOESM1_ESM.docx]

**Legends to Figures**

**Figure S1** DEGs intersection in GSE 9750, GSE 14404, GSE 63514, and GSE 63678 datasets venn diagram.

**Figure S2** Box diagram of mean age with high and low levels of gene expression groups in CEP55, MCM2, RFC4 and RRM2 genes.
