# Supplementary figures and images for: Identification of key biomarkers and related immune cell infiltration in cervical cancer tissue based on bioinformatics analysis
Source: Sci Rep. 2023 Jun 21;13:10121. doi: 10.1038/s41598-023-37346-z (PMC10284792; doi:10.1038/s41598-023-37346-z)

GSE63514

GSE14404

GSE63678

GSE9750

GSE7410

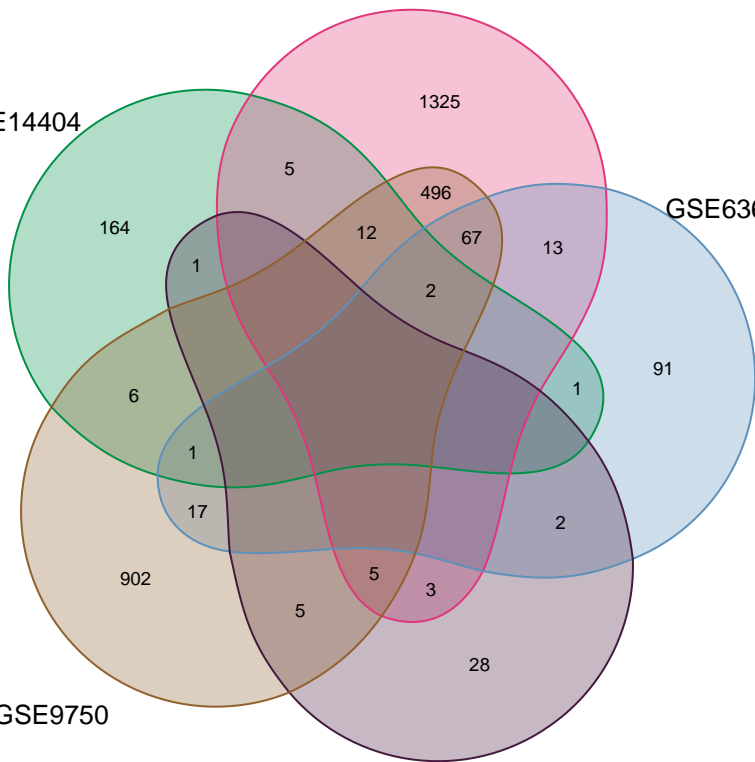

Supplement: Supplementary file 2 — Supplementary Figure S1. [file 41598_2023_37346_MOESM2_ESM.pdf]

## CEP55

## MCM2

Age

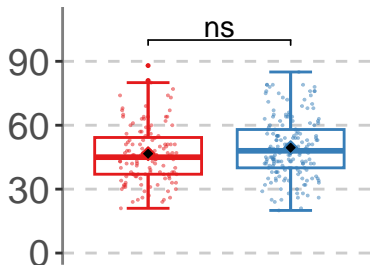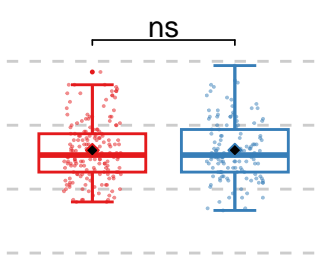

## RFC4

## RRM2

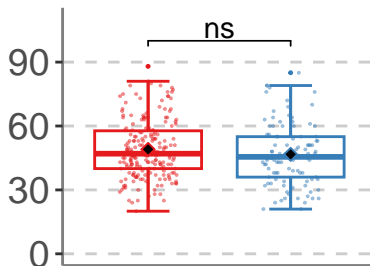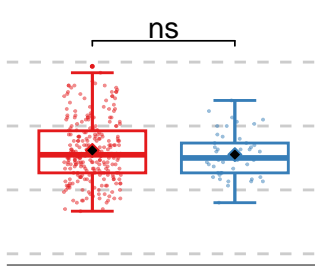

High

Low

High

Low

Supplement: Supplementary file 3 — Supplementary Figure S2. [file 41598_2023_37346_MOESM3_ESM.pdf]

**Table S2** Network module (1−3) analysis

| Number | Modular | Score | Spot | Edge |
| --- | --- | --- | --- | --- |
| 1 | 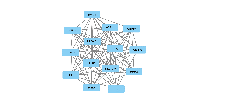 | 12.769 | 14 | 83 |
| 2 | 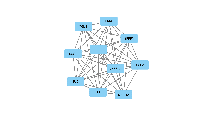 | 10 | 10 | 45 |
| 3 | 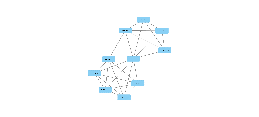 | 5.778 | 10 | 26 |

Supplement: Supplementary file 5 — Supplementary Table S2. [file 41598_2023_37346_MOESM5_ESM.docx]
