# Supplementary material for: Identification of key biomarkers and related immune cell infiltration in cervical cancer tissue based on bioinformatics analysis
Source: Sci Rep. 2023 Jun 21;13:10121. doi: 10.1038/s41598-023-37346-z (PMC10284792; doi:10.1038/s41598-023-37346-z)
Supplement: Supplementary file 4 — Supplementary Table S1. [file 41598_2023_37346_MOESM4_ESM.docx]

**Table S1** Relevant information from five groups of GEO CC data

| Chip experiment no. | Chip platform | Study population | Normal cervical samples | CC samples |
| --- | --- | --- | --- | --- |
| GSE7410 | GPL1708 | Netherlands | 5 | 21 |
| GSE9750 | GPL96 | USA | 15 | 32 |
| GSE14404 | GPL6699 | India | 5 | 28 |
| GSE63514 | GPL570 | USA | 24 | 28 |
| GSE63678 | GPL571 | USA | 5 | 5 |
