# Supplementary material for: Identification of key biomarkers and related immune cell infiltration in cervical cancer tissue based on bioinformatics analysis
Source: Sci Rep. 2023 Jun 21;13:10121. doi: 10.1038/s41598-023-37346-z (PMC10284792; doi:10.1038/s41598-023-37346-z)
Supplement: Supplementary file 6 — Supplementary Table S3. [file 41598_2023_37346_MOESM6_ESM.docx]

**Table S3** KEGG signal pathway analysis results of module 1−3

| Number | Modular | Signaling Pathway |
| --- | --- | --- |
| 1 | RRM2, SPAG5, TPX2, MELK, CENPE, TTK, DLGAP5, ASPM, NUSAP1, PRC1, TOP2A, KIF4A, CEP55, KIF20A | Pyrimidine metabolism  Glutathione metabolism  Platinum drug resistance |
| 2 | PPL, TGM1, DSG1, FLG, IVL, SPRR3, SPRR1A, SPRR1B, SPRR2B, DSG2 | Arrhythmogenic right ventricular cardiomyopathy  Staphylococcus aureus infection |
| 3 | CXCL8, CXCL10, CXCL13, CXCL1, CRISP3, CXCL9, MMP9, PRSS3, CXCL11, CDA | Viral protein interaction with cytokine and cytokine receptor  Chemokine signaling pathway  Cytokine−cytokine receptor interaction |
