# Supplementary material for: Identification of key biomarkers and related immune cell infiltration in cervical cancer tissue based on bioinformatics analysis
Source: Sci Rep. 2023 Jun 21;13:10121. doi: 10.1038/s41598-023-37346-z (PMC10284792; doi:10.1038/s41598-023-37346-z)
Supplement: Supplementary file 7 — Supplementary Table S4. [file 41598_2023_37346_MOESM7_ESM.docx]

**Table S4** Description of the 12 hub genes

| Gene | Full name | Alias | Function |
| --- | --- | --- | --- |
| KIF20A | kinesin family member 20A | MKLP2, RAB6KIFL, RCM6 | Mitotic kinesin required for chromosome passenger complex (CPC)-mediated cytokinesis |
| TPX2 | TPX2 microtubule nucleation factor | C20orf1, C20orf2, DIL-2, DIL2, FLS353, GD:C20orf1, HCA519, HCTP4, REPP86, p100 | Spindle assembly factor required for normal assembly of mitotic spindles |
| CENPE | centromere protein E | CENP-E, KIF10, MCPH13, PPP1R61 | Microtubule plus-end-directed kinetochore motor, which plays an important role in chromosome congression, microtubule-kinetochore conjugation, and spindle assembly checkpoint activation |
| CEP55 | centrosomal protein 55 | C10orf3, CT111, MARCH, URCC6 | Plays a role in mitotic exit and cytokinesis |
| TOP2A | DNA topoisomerase II alpha | TOP2; TP2A; TOPIIA; TOP2alpha | Key decatenating enzyme that alters DNA topology. May play a role in regulating the period length of ARNTL/BMAL1 transcriptional oscillation |
| FOXM1 | forkhead box M1 | MPP2; HFH11; HNF-3; INS-1; MPP-2; PIG29; FKHL16; FOXM1A; FOXM1B; FOXM1C; HFH-11; TRIDENT; MPHOSPH2 | Transcriptional factor regulating the expression of cell cycle genes essential for DNA replication and mitosis. Plays a role in the control of cell proliferation. |
| OIP5 | Opa interacting protein 5 | CT86; MIS18B; LINT-25; MIS18beta; hMIS18beta; 5730547N13Rik | Required for recruitment of CENPA to centromeres and normal chromosome segregation during mitosis |
| RRM2 | ribonucleotide reductase regulatory subunit M2 | R2; RR2; RR2M; C2orf48 | Provides the precursors necessary for DNA synthesis. Catalyzes the biosynthesis of deoxyribonucleotides from the corresponding ribonucleotides. Inhibits Wnt signaling. |
| RFC4 | replication factor C subunit 4 | A1; RFC37 | The elongation of primed DNA templates by DNA polymerase delta and epsilon requires the action of the accessory proteins proliferating cell nuclear antigen (PCNA) and activator 1 |
| GINS1 | GINS complex subunit 1 | PSF1; IMD55 | Required for correct functioning of the GINS complex |
| MMP9 | matrix metallopeptidase 9 | GELB; CLG4B; MMP-9; MANDP2 | Matrix metalloproteinase that plays an essential role in local proteolysis of the extracellular matrix and in leukocyte migration |
| MCM2 | minichromosome maintenance complex component 2 | BM28; CCNL1; CDCL1; cdc19; DFNA70; D3S3194; MITOTIN | Acts as a component of the MCM2-7 complex (MCM complex), which is the putative replicative helicase essential for “once per cell cycle” DNA replication initiation and elongation in eukaryotic cells |
